# State Greetings

**Published:** 1902-10

**Authors:** 


### STATE GREETINGS.

The New York State Society wants its sister organization in Pennsylvania to attend to its own business, as it does not want it known what they stand for over there outside the borders of its own Commonwealth.

It wants a special privilege to condone the acts of one of its members, an officer who teaches the false doctrine that tuberculosis may be engendered by bad sanitary conditions, because he is in the employ of a commercial milk aggregation.

It claims the special right to condemn a flagrant act of professional treachery unequalled in the history of veterinary progress at home or abroad, on the floor of the national organization, and then go home and condone the offence and apologize for hurting the feelings of one of their number.

It wants a special right given to a select few to establish State laws of such a character, under the cloak of higher veterinary education, that has practically strangled two of the best and oldest veterinary institutions in the land, and when, to avoid just criticism for their own failure, to import citizens from other States, implant them in their own Commonwealth to grow for three years to swell the roll of students.

It wants the privilege to dictate to other States that their laws shall be so arranged as to accept without question their offspring, and, when refused, to clandestinely libel the good name of members of a sister State Board in language it would not have the courage to write of them directly. Pennsylvania should not be alarmed at the attitude of their New York brethren, but continue to aid them by a just and well-merited criticism from time to time. It is wholesome and educational.

---

The Journal wishes the Chief of the Bureau of Animal Industry a pleasant trip abroad. We are assured of the profit that will accrue to our live-stock industries, the credit that will fall to our Bureau of Animal Industry, and the increased respect at home and abroad for our Department of Agriculture. To this department there has been no one man attached thereto who has brought more credit to its worth, maintained its good name, or rendered richer services to the interests it guards than Dr. D. E. Salmon, Chief of the Bureau of Animal Industry.

